# Alternative strategies for mosquito-borne arbovirus control

**DOI:** 10.1371/journal.pntd.0006822

**Published:** 2019-01-03

**Authors:** Nicole L. Achee, John P. Grieco, Hassan Vatandoost, Gonçalo Seixas, Joao Pinto, Lee Ching-NG, Ademir J. Martins, Waraporn Juntarajumnong, Vincent Corbel, Clement Gouagna, Jean-Philippe David, James G. Logan, James Orsborne, Eric Marois, Gregor J. Devine, John Vontas

**Affiliations:** 1 Department of Biological Sciences, Eck Institute for Global Health, University of Notre Dame, Notre Dame, Indiana, United States of America; 2 Department of Medical Entomology & Vector Control, School of Public Health, Tehran University of Medical Sciences (TUMS), Tehran, Iran; 3 Global Health and Tropical Medicine, GHTM, Instituto de Higiene e Medicina Tropical, IHMT, Universidade Nova de Lisboa, UNL, Lisboa, Portugal; 4 Environmental Health Institute (EHI), National Environment Agency (NEA), Singapore; 5 Instituto Oswaldo Cruz (IOC), Fundação Oswaldo Cruz (FIOCRUZ), Rio de Janeiro, Brazil; 6 Department of Entomology, Kasetsart University (KU), Bangkok, Thailand; 7 Institut de Recherche pour le Développement (IRD), Maladies Infectieuses et Vecteurs, Ecologie, Génétique, Evolution et Contrôle, University of Montpellier, Montpellier, France; 8 Centre National de la Recherche Scientifique (CNRS), Laboratoire d'Ecologie Alpine (LECA), Université Grenoble-Alpes, Domaine universitaire de Saint Martin d'Hères, Grenoble, France; 9 Department of Disease Control, London School of Hygiene and Tropical Medicine, London, United Kingdom; 10 ARCTEC, London School of Hygiene and Tropical Medicine, London, United Kingdom; 11 Université de Strasbourg, CNRS UPR 9022, INSERM U963, Strasbourg, France; 12 QIMR Berghofer Medical Research Institute, Brisbane, Australia; 13 Institute Molecular Biology and Biotechnology (IMBB), Foundation for Research and Technology (FORTH), Crete, Greece; 14 Pesticide Science Lab, Agricultural University of Athens, Athens, Greece; Faculty of Science, Mahidol University, THAILAND

## Abstract

**Background:**

Mosquito-borne viruses—such as Zika, chikungunya, dengue fever, and yellow fever, among others—are of global importance. Although vaccine development for prevention of mosquito-borne arbovirus infections has been a focus, mitigation strategies continue to rely on vector control. However, vector control has failed to prevent recent epidemics and arrest expanding geographic distribution of key arboviruses, such as dengue. As a consequence, there has been increasing necessity to further optimize current strategies within integrated approaches and advance development of alternative, innovative strategies for the control of mosquito-borne arboviruses.

**Methods and findings:**

This review, intended as a general overview, is one of a series being generated by the Worldwide Insecticide resistance Network (WIN). The alternative strategies discussed reflect those that are currently under evaluation for public health value by the World Health Organization (WHO) and represent strategies of focus by globally recognized public health stakeholders as potential insecticide resistance (IR)-mitigating strategies. Conditions where these alternative strategies could offer greatest public health value in consideration of mitigating IR will be dependent on the anticipated mechanism of action. Arguably, the most pressing need for endorsement of the strategies described here will be the epidemiological evidence of a public health impact.

**Conclusions:**

As the burden of mosquito-borne arboviruses, predominately those transmitted by *Aedes aegypti* and *A*. *albopictus*, continues to grow at a global scale, new vector-control tools and integrated strategies will be required to meet public health demands. Decisions regarding implementation of alternative strategies will depend on key ecoepidemiological parameters that each is intended to optimally impact toward driving down arbovirus transmission.

## Author summary

International public health workers are challenged by the burden of arthropod-borne viral diseases, to include mosquito-borne arboviruses transmitted by *Aedes aegypti* and *A*. *albopictus* due in part to lack of sustainable vector control and insecticide resistance (IR), as well as the inability to scale up and sustain existing interventions for prevention of urban epidemics. As a consequence, there has been increasing interest to advance the development of alternative methods. This review provides a general overview of alternative vector-control strategies under development for the control of arbovirus mosquito vectors and highlights how each could offer innovative public health value. Considerations to regulations, acceptance, and sustainability are also provided.

## Introduction

International public health workers are challenged by a burden of mosquito-borne arboviral diseases despite best efforts in control programs. An estimated 4 billion people live in areas at risk for dengue virus transmission alone [1]. Well-documented successes indicate that rigorously applied vector control using existing interventions can reduce arbovirus transmission and disease [2,3]; however, the degree to which such strategies may have prevented epidemics and the spread of arbovirus diseases is not well understood due to lack of evidence [4]. Despite existing interventions, epidemics and spread of arbovirus diseases continue. The reasons for this are complex but include inadequate program implementation; ineffective coverage; lack of human, financial and infrastructural capacity; insecticide resistance (IR); political will; and inability to scale. Integrated approaches and advancements in development of alternative strategies have been of renewed focus. This review provides an overview of strategies under development for the control of arbovirus mosquito vectors, focusing primarily on *Aedes aegypti* and *A*. *albopictus*.

## Rationale for developing alternative strategies

A primary strategy for arbovirus outbreak control, such as dengue, is the use of synthetic chemicals with quick-action killing of adult vectors using space spraying [2,5,6]. The majority of recommended insecticides are of the pyrethroid chemical class, creating challenges to preventing selection pressure on susceptible mosquito populations as well as the control of pyrethroid-resistant vectors [5]. Regarding arbovirus vector population management, specifically of *A*. *aegypti*, larval control has long been proposed and implemented as a primary strategy [7], including applications of chemical and microbial larvicides, insect growth regulators (IGRs), and bacterial toxins [8]. Biological agents used against immatures include predatory copepods, fish, and *Toxorhynchites* larvae. Arguably, the greatest obstacle to *A*. *aegypti* larval control success is dependency on the ability to detect, access, and eliminate or treat domiciliary—often cryptic—breeding sites, a challenging and costly task that often leads to low coverage. In addition, their reduced efficiency in some occasions limits their widespread adoption [9].

## Outlook on alternative strategy development

**Fig 1:**
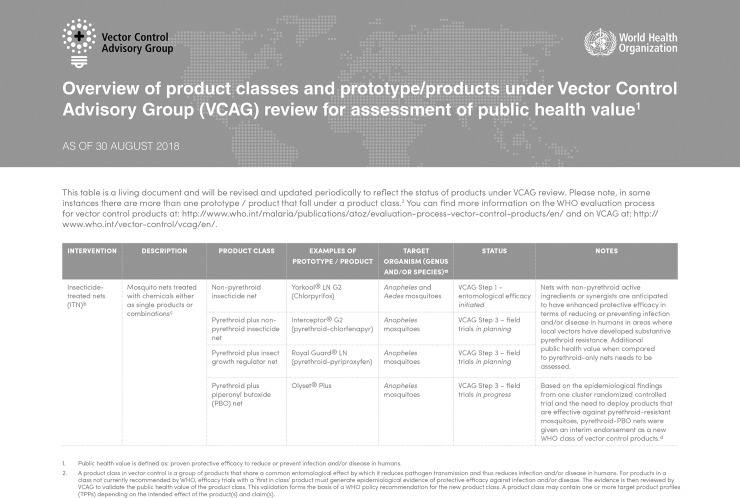
WHO VCAG overview of new vector control product classes and status of evaluation. Available from: http://apps.who.int/iris/bitstream/handle/10665/274451/WHO-CDS-VCAG-2018.03-eng.pdf?ua=.

New tools are being developed on the premise that significant health benefit can be demonstrated in at least two endemic settings, aiming at niche roles rather than becoming the default intervention across a wide range of settings. Priority is given to tools that will improve current interventions in areas where they are challenged, either due to vector behaviors that prevent mosquito interaction with the intervention, IR, and/or residual disease transmission [5]. Several new strategies and product classes are under review by the World Health Organization Vector Control Advisory Group (WHO VCAG) [10] (Fig 1).

We have used the current VCAG portfolio as a basis for our “inclusion criteria” for interventions described in this review. Specifically, we review strategies for which evaluations (1) have previously been conducted against arbovirus mosquito vectors demonstrating evidence of entomological success (e.g., autodissemination using entomopathogenic fungi or pyriproxyfen, pyrethroid treated traps, attractive targeted sugar baits [ATSB], *Wolbachia*, genetic manipulation) and/or (2) are actively underway against arbovirus vectors (e.g., spatial repellents, treated materials, sterile insect technique [SIT]). We did not include strategies currently being evaluated only against anopheline vectors (e.g., insecticide-treated eave tubes). The exception is gene drive due to historical theory and/or evaluations of entomological impact, expectation of broad utility of this strategy across disease vectors, and potential to overcome challenges posed by traditional genetic manipulation. In addition, the VCAG met in Geneva in November 2016 to review new potential vector control based on “genetic manipulation of mosquitoes through gene drive technology to reduce vector populations and transmission”; therefore, gene drive is part of the portfolio of vector control tools (VCTs) under consideration by WHO [11].

WHO has formally recognized some of these strategies for arbovirus control and encouraged testing in affected countries following appropriate monitoring and evaluation procedures [12]. Among the VCAG criteria for facilitating WHO recommendation, arguably the most critical is epidemiological evidence to endorse full-scale implementation. Funding to support rigorous pilot trials for generating preliminary evidence supportive of large-scale clinical trials as well as for randomized cluster trials themselves must be forthcoming. Where funding is not made available, support for delivering the requisite evidence base by other means, such as analyses of historical data captured during public health exercises, must be advocated. Other VCAG criteria include entomological correlates of protection, acceptability, and manufacturability (Table 1).

**Table 1:** Example epidemiological−entomological parameters intended to demonstrate public health value of a new vector-control tool^1^.

Parameters	Requirements
Epidemiology	A significant reduction in incidence of pathogen infections compared to control using randomized cluster trial. Level of compliance and coverage required in relation to efficacy detected.
Entomology	Trends indicating significantly reduced vector population density, longevity/population age structure (parity rates), and/or arbovirus infection over time between treatment arms.
Economics	Projected cost per unit protected similar to, or less than, currently deployed arbovirus vector-control strategy in trial setting.
Technology development	Prototype or product is essentially ready to manufacture at scale; may require minor changes to improve the method in response to trial outcomes.
Manufacturability sustainability	Confirmation of commercial sustainability by manufacturer/producer; early manufacturing/production runs at volume; intellectual property issues resolved and commercial production possible. Product procurements and pre- and post-marketing QA
User compliance/acceptability	User acceptability/compliance estimated.
Delivery and feasibility of implementation	Feasibility of intervention implementation demonstrated.
Regulatory/safety/ethical and environmental impact	Adverse events monitored during trial; registration of product.

^1^Adapted from the WHO VCAG (http://www.who.int/neglected_diseases/vector_ecology/Operational_procedures_for_VCAG.pdf?ua=1)

**Abbreviations:** QA, quality assurance; VCAG, Vector Control Advisory Group; WHO, World Health Organization.

## Alternative strategies

### Novel larvicides and applications: Entomopathogenic fungi, pyriproxyfen, and autodissemination

Entomopathogenic Ascomycetes fungi, especially *Metarhizium anisopliae* and *Beauveria bassiana*, have been suggested for control of both larval and adult stages of dengue vectors [13]. Fungal longevity (duration of efficacy once applied) may be one obstacle, with another being delivery of killing dose to target insect; therefore, formulation optimization is critical. Additionally, entomopathogens have fared badly in agriculture because they simply cannot compete with the costs and efficacy of chemical insecticides. This may change as insecticide regulation becomes more difficult and IR begins to dominate.

An approach to circumvent the difficulty of locating and treating immature habitats is autodissemination, wherein dispersal and transfer of actives is carried out by contaminated adult mosquitoes [14]. Contamination can occur through treated materials [15] or dissemination stations such as modified ovitraps [16,17]. Once contaminated, mosquitoes disperse the agent in subsequent contacts with untreated surfaces. Autodissemination can exploit polygamic behavior whereby treated males can contaminate multiple females during mating [18]. It is important to note that there must be amplification in coverage between the lure and aquatic habitat to achieve benefit beyond killing offspring of only contaminated adults.

An efficacy trial recently conducted in the United States showed a decline of *A*. *albopictus* populations following the field release of males contaminated with pyriproxyfen compared to untreated field sites [18], and the combination of pyriproxyfen autodissemination with the SIT (see below), a concept termed “Boosted SIT,” is being explored [19]. Pilot interventions with pyriproxyfen have given promising results [16,17,20]. Design of new types of dissemination stations and other actives [21] as well as new formulations (e.g., IGRs in combination with bacterial toxins of spinosad or fungi *B*. *bassiana*) may further improve effectiveness of autodissemination. Despite growing evidence of entomological efficacy, data requirements to demonstrate public health value are lagging (Table 1).

### Spatial repellents

Spatial repellents are products designed to release volatile chemicals into an air space, induce insect behavior modification to reduce human-vector contact, and thereby reduce pathogen transmission [22]. A spatial repellent product category is currently in Stage 3 of the VCAG assessment scheme, establishing proof-of-principle of efficacy through clinical trials (Fig 1). The application of spatial repellents at the household level through a consumer product market offers a “bottom-up” user-centric strategy for enhanced uptake (coverage), potentially overcoming challenges of scale [23]. Evidence that community-led campaigns can impact transmission is needed. Spatial repellents may also be implemented through a donor-subsidized market similar to that used for insecticide-treated bed nets against malaria.

Studies have demonstrated that chemicals currently recommended for vector control can elicit varied responses dependent on concentration [24]. For example, pyrethroids used in space spraying are applied at predominantly toxic levels to kill mosquitoes that land on treated surfaces or through contact with forced dispersal of formulated droplets. Other pyrethroids—labeled spatial repellents—such as transfluthrin and metofluthrin, are highly volatile at ambient temperature and disperse passively to repel, inhibit host-seeking, or kill mosquitoes depending on the chemical concentration gradient in the air space [25,26]. A range of spatial repellent products has demonstrated reduction in human−vector contact, and coils have been shown to contribute to reduction in human malaria infection [27,28]. Historically, the mode of action (MoA) of spatial repellent products has been focused on “movement away from the chemical source without the mosquito making physical contact with the treated surface” (deterrency), an expanded concept that reflects the complexity in defining spatial repellents, includes chemical actions that interfere with host detection and/or disrupt blood-feeding, and was established by WHO in 2013” [29]. Knowledge gaps exist about how spatial repellents work, including exact molecular and physiological mechanisms [30], the hereditary basis by which spatial repellent traits are maintained in populations, and the relationship of response intensity with IR [31]. All are vital characterizations required in discovery and optimization of spatial repellent compounds and formulated products.

Despite this, demonstration of efficacy in pyrethroid-resistant *A*. *aegypti* [32], as well as enhanced *A*. *aegypti* attraction response of gravid females to experimental ovipostion sites following exposure [33], may offer insights into complementary or synergistic roles for spatial repellents in integrated vector management strategies.

### Traps

Mosquito traps have served for decades as effective surveillance tools but have only recently been considered under VCAG as a control strategy (Fig 1). For a trap to be an efficient tool for vector elimination, it must be highly sensitive and specific for a target species. The most effective traps rely on a combination of attractant cues such as light, heat, moisture, carbon dioxide, and synthetic chemicals for host attraction [34]. The Centers for Disease Control and Prevention (CDC) miniature light trap was introduced in 1962 [35], but major advances have been made with newer traps that have incorporated innovations such as carbon dioxide plumes and counter flow geometry [36]. Another trap recently developed is the Mosquito Magnet Pro (MMPro), which is commercially available and uses propane, making it affordable and easily deployable for the general public.

Expanding on the trap concept for arbovirus mosquito vector population reduction was the introduction of the lethal ovitrap. These traps are designed to attract and kill egg-bearing females. The attractive baited lethal ovitrap (ALOT) has shown promise in both laboratory and field settings for significantly reducing *Aedes* populations. A prospective nonrandomized field trial of the ALOT trap was conducted in two cohorts of Iquitos, Peru. One year into the trial, dengue incidence as measured by febrile surveillance was 75% lower in the intervention area compared to the control cohort [37]. Although there have been a number of unsuccessful attempts to document significant reductions in vector densities using lethal ovitraps, it appears that sufficient coverage with an appropriate number of traps per unit area is key to this strategy’s success [38]. Other research has demonstrated potential of the baited gravid (BG)-Sentinel trap as a vector-control tool [39]. Studies with area-wide use of autocidal gravid ovitrap (AGO) in Puerto Rico have shown 80% reduction in densities of female *A*. *aegypti* for up to 1 year [40]. In Brazil, significant reductions in densities of gravid *A*. *aegypti* by the biogents passive gravid *Aedes* trap (BG-GAT) [39] was achieved.

Incorporating a distribution model of peridomestic lethal ovitraps to remove gravid females from the vector population is anticipated to complement current *A*. *aegypti* control campaigns focused on source reduction of larval habitats. As naturally preferred oviposition sites are removed from the environment, artificial traps may become more effective at reducing vector populations; however, routine monitoring and/or retrieving of traps, if no longer used, must be incorporated to avoid traps becoming potential breeding sites in scaled control programs. In anticipation of success based on this concept, WHO has currently developed guidelines for efficacy testing of traps against arbovirus vectors to include indicators [http://apps.who.int/iris/bitstream/handle/10665/275801/WHO-CDS-NTD-VEM-2018.06-eng.pdf?ua=1].

### ATSB

The ATSB product class is currently at Stage 2 of the VCAG evaluation pathway (Fig 1). The success of an ATSB strategy relies on attracting mosquitoes to and having them feed on toxic sugar meals sprayed on plants or used in bait stations. The use of sugar feeding to reduce mosquito populations was first reported by Lea in 1965 [41] and then other studies [42–49] in *A*. *albopictus*, other culicines, and sand flies.

ATSBs for both indoor and outdoor control of mosquito vectors may impact populations by direct mortality induced by feeding on an insecticide bait, and/or, dissemination through a bait of mosquito pathogens or nonchemical toxins [50]. Because both female and male mosquitoes require sugar throughout the adult lifespan, the potential effects of this intervention on a vector population may be dramatic but will depend on feeding behavior (e.g., readiness to sugar feed indoors). Bait solutions are composed of sugar, an attractant, and an oral toxin. Toxins tested include boric acid [51], spinosad, neonicotinoids, and fipronil [52], as well as several other classes of insecticide [49]. Some attractants are focused on locally acquired sugars, juices, and fruit as mosquitoes may be selective toward carbohydrates originating from their geographic range, although more “general” attractants have been developed more recently [48,53].

A cumulative effect of ATSBs on an anopheline mosquito population was demonstrated for an area with alternative sugar sources resulting in delayed population level lethality as compared to sugar-poor areas [54], suggesting its utility in arid environments. A single application of an ATSB affected *Anopheles sergentii* density, parity, survival, and hence vectorial capacity [54]. Potential drawbacks of this strategy might be the effects on nontarget sugar-feeding organisms, as well as the high coverage required. Environmental, safety, and cost estimates are being explored as part of the WHO VCAG requirements for demonstration of public health value (Table 1).

### Insecticide-treated materials

Insecticide-treated materials (ITMs) can provide bite protection by killing or repelling vectors. ITMs, in the form of clothing, can be worn outside during the day, at work, or in school, offering protection where current mosquito control strategies, such as bed nets, may not. Military and other commercial companies have used insecticide-treated clothing to protect their workers from biting arthropods, and ITMs have reduced the incidence of vector-borne diseases such as malaria and leishmaniasis [55].

Currently, an ITM strategy for vector control is in Stage 1 of VCAG evaluations (Fig 1), with permethrin (a pyrethroid) being the only active ingredient used in ITMs due to requirements of meeting human safety profiles [55]. Permethrin ITMs have demonstrated personal protection against *A*. *aegypti* mosquitoes in various laboratory experiments [56–58], reduction of *A*. *aegypti* human biting rates by 50% (partial limb coverage) to 100% (when fully clothed) in semifield trials [57,58], and an 80% reduction in *A*. *aegypti* densities after just one month of children wearing permethrin treated school uniforms [59]. Models have estimated permethrin-treated uniforms could reduce dengue infections by up to 55% in the most optimistic scenarios [60].

Despite the potential for ITMs to control arbovirus vectors, current application techniques and formulations have limited efficacy under general use as permethrin washes out of material after several washes and is degraded by UV and heat exposure [56,61]. Novel formulations are needed to achieve long-lasting, effective release of permethrin under anticipated use. A technology being developed to address this challenge is microencapsulation, which binds deeper within the fabric and increases insecticide stability, allowing for a more consistent and extended release of the active ingredient [62]. Novel active ingredients that have far-ranging efficacy compared to permethrin and/or represent a different chemical class (including natural ingredients) will be needed to overcome biting on exposed skin not covered by ITMs, as well as to address pyrethroid resistance, respectively [63].

As with other alternative strategies under evaluation, it will be imperative that epidemiological evidence be generated in robust trial designs before wider implementation of ITMs can be recommended. Efficacy of ITMs will be dependent on user compliance; therefore wearable technologies must be acceptable to target populations (see “Acceptability and compliance of alternative methods” section).

### Classical SIT

The sterile insect technique (SIT) is based on the release of sterilized male insects, traditionally by means of irradiation, to suppress vector mosquito populations. SIT induces random lethal dominant mutations in the germ cells, which acts on the eggs in the female to prevent fertilization [64]. The concept is that sterile males will mate with wild females without producing any offspring. A major challenge to scale implementation has been building infrastructure in endemic settings to support mass rearing of the target vector. New technologies for mass rearing of mosquitoes, especially *Aedes*, are currently available [65–67]; however, improvement in the sterilization process is still needed to avoid somatic damage, resulting in reduction of longevity, problems with sexual vigor, and overall male activity [68]. Although encouraging results of SIT have been obtained with *A*. *albopictus* [69], operational cost still constitutes a significant barrier to large-scale rearing facilities in endemic countries.

### Release of insects with dominant lethality

The release of insects with dominant lethality (RIDL) strategy reduces vector populations (self-limiting approach) through individuals carrying a transgenic construct, which acts on the late larval stage and the pupae to prevent survival to imago. In contrast to both SIT and *Wolbachia*-based population suppression (see “Gene drives” section), for RIDL technology, eggs must become fertilized for subsequent impact. The engineered effector gene is homozygous, repressible dominant lethal, and activates its own promoter in a positive feedback loop but can be regulated using an external activator. The construct also includes a reporter gene resulting in RIDL insects expressing a visible fluorescent marker for easy screening of transgenic and hybrid individuals before and after an intervention [70].

RIDL transgenic constructs have been successfully integrated into *A*. *aegypti* laboratory strains by Oxitec,which has performed open release trials with the OX513A strain since 2009 in Brazil, Cayman Islands, and Panama, with approvals pending for trials in the US and India. In Brazil, Panama, and Cayman, wild *A*. *aegypti* populations were reduced by more than 90% after release of the OX513A strain [71,72]. The sustainability of this reduction will depend on methods to avoid a new population increase from the remaining insects, hatching of dried eggs, and migration from uncontrolled areas. A continued monitoring system will provide a better overview of the impact of this method in long-term suppression of *A*. *aegypti*.

Although the RIDL strategy has advanced to VCAG Stage 3 (Fig 1), a concern was that the reduction of *A*. *aegypti* could favor its replacement by *A*. *albopictus*. It should be noted that suppression of a population by any technique might encourage invasion and replacement by competitors. In Panama, however, six months after the OX513A *A*. *aegypti* releases ceased, there was no evidence of either expansion or augmented density of *A*. *albopictus* where both species occurred in sympatry [72].

### Wolbachia

*Wolbachia* is a natural intracellular bacterial symbiont found in at least 60% of insects known to alter reproduction of its host. Present in the female germline of an infected insect, *Wolbachia* is maternally transmitted to offspring. It can induce cytoplasmic incompatibility (CI), where mating between *Wolbachia*-infected males and uninfected females yields eggs that fail to develop. Regular releases of male mosquitoes infected with a *Wolbachia* strain not present in the wild-type mosquito could theoretically reduce the viability of eggs in the field and lead to population suppression. Alternately, *Wolbachia* strains may cause a decrease in the vectorial capacity of the vector (pathogen interference)—directly by interfering with competence or indirectly by shortening lifespan [73]. The wMel *Wolbachia* strain currently being assessed by VCAG, in Stage 3 of evaluations, is intended for population replacement that interferes with ability to transmit pathogens (Fig 1).

Regarding population suppression, though it was reported almost 50 years ago that *Culex pipiens fatigans* was eradicated via CI in a village in Myanmar (then Burma), it was only in 1971 that the involvement of *Wolbachia* was reported [74]. Successes in species-specific suppression using inundative male releases have been demonstrated in semifield and full-field trials involving *Aedes polynesiensis* in French Polynesia [75] and *A*. *albopictus* in Lexington, Kentucky, US [76]. Releases involving *Wolbachia*−*A*. *aegypti* have also started in California, Thailand, Singapore, and Australia. The safety of the approach has been thoroughly evaluated and reported [77]. Although results of field studies have been promising, full-scale sustainable deployment of a *Wolbachia* strategy requires more developmental efforts. Besides the need for an arsenal of *Wolbachia* vector strains that offers excellent CI, there is a need for active community engagement to gain public acceptance and a need to scale up the production of large numbers of *Wolbachia* mosquitoes through automation and optimization.

The population replacement strategy is based on the capacity of *Wolbachia* to invade and persist in wild mosquito populations, decreasing their vector competence [78]. Advantages of a replacement strategy, at its full potential, is the lack of requirement for consistent and continuing releases or reliance on community engagement to achieve near universal coverage. In the case of *A*. *aegypti*, the *Wolbachia* evaluation of a population replacement strategy has achieved important goals, such as a fruit fly *Wolbachia* strains can invade and sustain themselves in mosquito populations, reduce adult lifespan, affect mosquito reproduction, and interfere with pathogen replication [79]. So far three *Wolbachia* transinfection strains have been used in *A*. *aegypti*: w*AlbB* [80] introduced from *A*. *albopictus*, and *wMel* and w*MelPop* from *Drosophila melanogaster* [81]. The w*Mel Wolbachia* strain has the ability to reduce *A*. *aegypti* vectorial capacity to dengue and chikungunya viruses [81,82], an encouraging result recently extended to Zika virus, as indicated by experimental infection and transmission assays in *w*Mel-infected mosquitoes [83].

The World Mosquito Program aims to promote research in arbovirus control by releasing *Wolbachia*-infected *A*. *aegypti* in dengue-affected communities (www.eliminatedengue.com). The first release occurred in Australia in 2011. Two natural populations were successfully invaded after 10 weekly releases, nearly reaching fixation five weeks after releases stopped [84]. This high infection rate has been maintained through 2017 without further input. The substitution of a natural *A*. *aegypti* population by a w*Mel*-infected *A*. *aegypti* one prompted a scale up to other countries, including Vietnam, Indonesia, Brazil, and Colombia.

### Gene drives

Gene drives are transgenic constructs that possess the property to invade populations of the target species, even when conferring a fitness cost. The concept applied to mosquito control was proposed by Austin Burt as early as 2003 [85] and has since been a topic of research to spread a desired trait in mosquito species.

**Fig 2:**
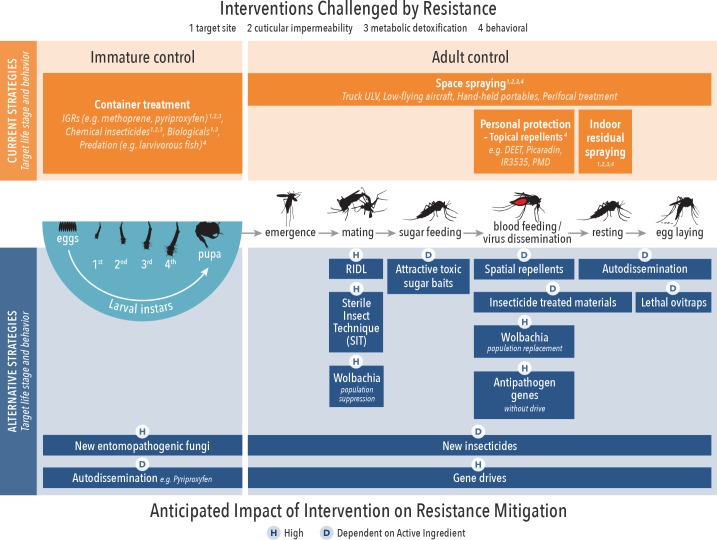
Principle of a gene drive. (A) Initial integration of a gene drive construct into the mosquito genome: Cas9 and the gRNAs encoded in the transgenic construct prepared as a plasmid can serve as molecular scissors mediating their own integration into the genomic target site they cut. Asterisks represent the cut sites determined by the gRNAs (three gRNAs in this example). Homologous recombination-mediated knock-in of the transgenic cassette occurs thanks to the target site flanking sequences cloned into the plasmid. (B) Spread of the gene drive in a mosquito population: mating between transgenic and nontransgenic mosquitoes places the transgenic construct in the presence of wild-type chromosomes that get cut by Cas9 at the target site determined by the gRNA(s). This break is repaired most frequently by homologous recombination with the intact chromosome, effectively copying the *trans*-gene into the broken wild-type chromosome and converting a heterozygous into homozygous cell. Cas9, CRISPR associated protein 9; gRNA, guide RNA.

Current gene drive designs are based on the Clustered Regularly Interspaced Short Palindromic Repeats—CRISPR-associated protein 9 (CRISPR-Cas9) system. The transgenic element must be inserted precisely in the sequence that it is designed to cleave. To achieve this, a “cassette” is integrated by “gene knock-in” [86]. The drive cassette thus becomes heritable and able to “drive” (Fig 2). Alternatively, docking sites can be knocked in the gene of interest and subsequently serve as acceptors for the gene drive cassette [87].

Two strategies are under evaluation in VCAG Step 1 based on gene drives—population replacement and population suppression (Fig 1). In the latter, a drive element can be designed to insert into and inactivate a sex-specific fertility gene, suppressing the population as the resulting “sterility allele” increases its frequency. This approach was illustrated by a recent proof-of-principle laboratory study in the African malaria vector *Anopheles gambiae* [87] and could rapidly be adapted to *A*. *aegypti* and *A*. *albopictus*. Gene drives may prove to function less efficiently in the field than in the laboratory [88] due to the selection of pre-existing refractory variants in the guide RNA (gRNA) targets. Should gene drives function almost as efficiently in the field as in theory, the suppression approach carries the potential to eradicate the target species altogether, although insect population substructuring would probably allow the existence of residual pockets of intact populations [89,90].

Gene-drive designs for population suppression are not yet optimal for release in the field; a female sterility-spreading gene drive will only function optimally if heterozygous females are fully fertile, which is not the case of the published designs. Optimized promoters that restrict Cas9 activity to germline tissues, or Cas9 variants with shorter half-lives, are avenues to explore to alleviate these limitations. A careful evaluation of the ecological impact of species eradication must be considered. Gene-drive animals represent a new class of genetically modified organism (GMO), the safety of which will need to be evaluated according to new criteria compared to traditional GMOs. This and other considerations, such as horizontal transfer, are the focus of a recent US National Academy of Sciences report on gene drives [91].

Gene drives can also be applied in population replacement strategies—using a drive construct to confer mosquito resistance to a given pathogen resulting in a population that becomes pathogen resistant as the genetic invasion progresses. Proof-of-principle has been demonstrated in the laboratory using the Asian malaria vector *Anopheles stephensi* [92]. In *Aedes*, antiviral constructs could be designed similarly, targeting one or several viruses. Candidate antiviral factors include RNA interference (RNAi) constructs, overexpressed components of the antiviral response, or RNA-targeting molecular scissors such as the recently identified CRISPR-Cas-like system C2c2 [93]. In all cases, it will be important to ascertain increasing resistance to a given virus does not render mosquitoes more susceptible to another.

### Other alternative strategies outside the VCAG portfolio

There are other product categories that have received recent attention but are in early development and not under VCAG assessment to include acoustic larvicides [94] and RNAi [95].

## Considerations for introducing alternative strategies

### Resistance management potential

IR in arbovirus vectors, such as *A*. *aegypti*, is considered a major obstacle to successful control [5]. The incidence of IR has increased rapidly in recent years [96], and concerns regarding environmental impact caused from insecticide residues continue [97]. This highlights the need for alternative methods to better manage arbovirus vector populations while mitigating selection pressure on existing IR genes [98]. Other WIN reviews will describe IR distribution, mechanisms, and management; we present here complementary considerations on the integration of alternative strategies for resistance. The use of nonchemicals, or chemicals with completely different MoAs (i.e., alternate target site), will potentially have a greater impact on insecticide resistance management (IRM), such as using IGRs for larval and pyrethroids for adult *A*. *aegypti* control, respectively [99]. Combination or rotations of unrelated compounds can (in theory) mitigate the occurrence of resistance and/or delay the selection process if already present at low levels. This may not hold true when considering metabolic resistance mechanisms by which one enzyme degrading one insecticide with a particular MoA may also degrade another insecticide with a different MoA. Reciprocally, two insecticides with the same MoA may not be degraded by the same enzymes (no cross metabolic resistance). The authors acknowledge that any tool is vulnerable to resistance development; therefore, robust monitoring and evaluation should accompany implementation.

**Fig 3:**
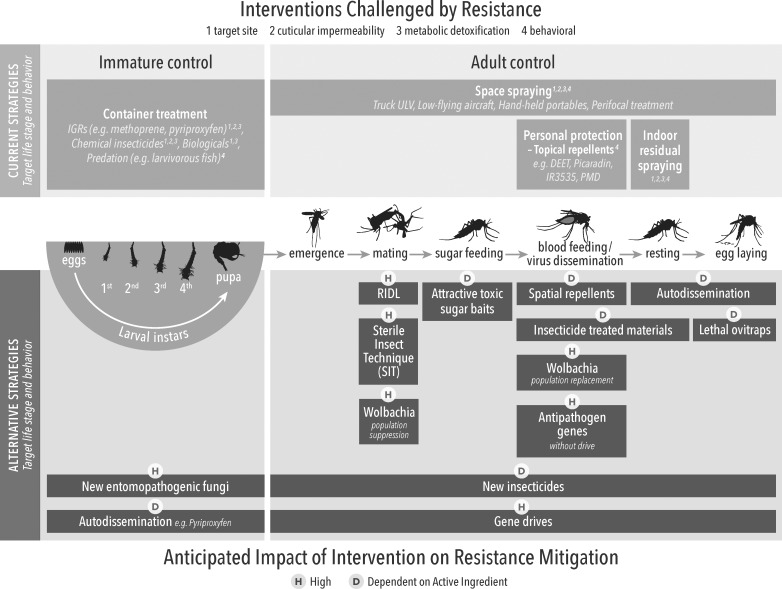
Current and alternative arbovirus control methods in the context of the targeted life stage of implementation and anticipated impact on IRM. IGR, insect growth regulator; IRM, insecticide resistance management; RIDL, release of insects with dominant lethality; ULV, Ultra-low volume spraying].

The potential of example alternative strategies to impact IRM is indicated in Table 2, whereas details of resistance risks are presented in Fig 3. There are a number of ways in which alternative strategies can control insecticide-resistant vectors, including different MoAs or different application methods (i.e., oral versus contact). Tests are available to measure and predict this "antiresistance" potential at several stages of product development; however, evidence is missing, for example, on the impact of target site resistance to pyrethroids on efficacy of transfluthrin (a pyrethroid spatial repellent) to reduce human infections in settings with resistant vector populations, or the efficacy of pyriproxyfen against insects overexpressing cytochrome P450s capable of metabolizing pyrethroids. In addition to cross resistance and efficacy assessments against pyrethroid-resistant mosquitoes, the introduction of novel active ingredients into VCTs requires careful monitoring of possible resistance development.

**Table 2:** Summary description of alternative vector-control tools, primary challenges, and benefits to include probability of mitigating evolutionary response/impact in resistance management.

Product category (strategy)	MoA^1^	Prototype product description	Intended application in operational deployment; primary challenge to success; primary benefit of operational deployment	Impact in resistance management based on MoA
**Novel larvicide approaches**	S/L	IGRs	Outdoors in immature habitats; presence of cryptic habitats; traditional deployment strategy	High
	S/L	Microbial insecticides (*Bacillus thuringiensis israelensis*	Outdoors in immature habitats; presence of cryptic habitats; traditional deployment strategy/aerial spray	High
	S/L	Entomopathogenic fungi	Indoors and outdoors; lethal effects to nontargets; can be applied on various surfaces	High
**Spatial repellents**	C	Passive emanator to reduce human−vector contact	Indoors and outdoors; resistance to currently available actives; can be delivered using consumer-product channels	Dependent on target site of actives
**Traps**	S	Captures and removes host seeking and/or gravid females	Indoors and outdoors; bulk; affordable and easy to use	Dependent on target site of actives
**ATSBs**	S/L	Attract and kill females and males (of all physiological status)	Outdoors; lethal effects to nontargets; easy to use	Dependent on target site of actives
**ITMs**	C	Clothing, blankets, screens, curtains with insecticides or spatial repellents	Indoors and outdoors; acceptability/behavior change by end-user; mobile technology	Dependent on target site of actives
**Classical SIT**	S/L	Release of radiated sterile male insects to sterilize females	Indoors and outdoors; colony maintenance, multiple releases, mating competitiveness; no nontarget effects	High
**RIDL**	S	Release of transgenic insects with dominant lethal construct to eliminate female progeny production	Indoors and outdoors; colony maintenance, multiple releases; no nontarget effects	High
***Wolbachia***	R	Population replacement (vectorial capacity)	Indoors and outdoors; colony maintenance, multiple releases, mating competitiveness; regulations for release	Moderate
	S/L	Population suppression (CI)	Indoors and outdoors; colony maintenance, multiple releases, mating competitiveness; no nontarget effects	High
**Gene drives**	R	Population replacement (introduction and spread of pathogen effector gene)	Indoors and outdoors; off-target effects (molecular); rate of spread through population	Low
	S/L	Population suppression (introduction and spread of lethal gene)	Indoors and outdoors; off-target effects (molecular); rate of spread through population	High

^1^Primary Entomological MoA: S, population suppression; R, population replacement; L, reduction of mosquito longevity and density; C, reduction of human-vector contact.

**Abbreviations:** ATSB, attractant toxic sugar baits; CI, cytoplasmic incompatibility; IGR, insect growth regulators; ITM, insecticide-treated materials; MoA, mode of action; RIDL, release of insects with dominant lethality; SIT, sterile insect technique.

Strategies used for IRM and the introduction of new VCTs is adopted from agricultural practices [100], for which another WIN review is forthcoming. IRM should be applied within integrated vector management (IVM) approaches [101], which is defined as the rational decision-making process for the optimal use of resources for vector control [102] and includes the use of multiple complementary tools. An important point to stress is that a novel vector-control product may be more useful via its role in IRM in a certain setting with high levels of resistance risk, even if it is of a similar efficacy to existing interventions (“non inferior”).

## Knowledge base on mosquito biology and behavior

The importance of characterizing the biological profile (genetic structure, insecticide susceptibility) of the local vector population should be considered in the context of IVM and IRM using alternative strategies. For instance, the first release of *Wolbachia* in Rio de Janeiro, Brazil, did not succeed, because the spread of the bacteria was not self-sustained. Although *w*Mel *Wolbachia* was already hosted by around 60% of the local *A*. *aegypti* after 20 weeks of insertion, this frequency rapidly dropped when releases stopped. This may likely have occurred because the released w*Mel*-infected *A*. *aegypti* strain was susceptible to pyrethroids, the class of insecticide largely used by residents in dwellings. It was known that the natural *A*. *aegypti* population of that locality was highly resistant to pyrethroids. When a new colony of *A*. *aegypti*, as resistant as the field population, was infected with w*Mel* and released in the field for 24 weeks, 80% of local *A*. *aegypti* presented the bacteria, and this index is continually increasing [103,104]. Additional characterizations of importance include male feeding behavior for optimizing ATSB and genotyping key phenotypes to identify targets for gene drives.

## Acceptability and compliance of alternative methods

Social science, effective training of staff, and capacity building have major roles in the deployment of novel VCTs. New interventions must be acceptable to the communities in which it will be used. One possible barrier is that homeowners might perceive the intervention as ineffective or harmful to themselves or their environment, and adoption of the strategy might be compromised. For example, populations may be reluctant to use juvenoids (e.g., pyriproxyfen) because the insecticide has a late killing action on pupae, leaving live larvae in the container. Regardless of how efficacious a control strategy may prove to be during proof-of-concept, it is critical to assess the potential barriers and/or acceptance of local populations in which implementation trials may occur early in the development phase, even within a limited participant population [105,106]. More robust surveys can be used once an intervention prototype is available [107]. Such surveys can be used to assess factors associated with adoption and maintenance behaviors and identify barriers to its correct and consistent use to ensure (or improve) product sustainability.

## Regulatory considerations for alternative control strategies

Regulatory considerations are required early in the development process to ensure data requirements are met for safety and use (Table 1). If a tool under investigation does not fall under an existing product category (i.e., is a new class), a new regulatory framework and/or data requirements may be warranted. It is important to note, regulation can be as substantial an obstacle to an effective intervention as any gap in research and development (R&D). Where regulations are prohibitive, options for arbovirus control will become even more constrained; this may be most evident when a lack of expertise and/or infrastructure exists around the new tool (e.g., biotechnology and gene drives).

Historically, the WHO Pesticide Evaluation Scheme (WHOPES) was the body responsible for review of product evaluation and recommendations [108]. However, a reform in the regulatory process for evaluation of new products has been made with the recent agreement by WHO to adopt the Innovation to Impact (I2I) initiative in 2017 [109]. The I2I is expected to accelerate the evaluation process, increase transparency, and improve quality assurances, similar to the framework already adopted for drug and vaccine prequalifications [110]. Development of new guidelines or modifications of existing ones for efficacy testing of alternative VCTs are anticipated to be needed as novel MoA are exploited. Likewise, if the formulated product under investigation is not registered in the study area for which it will be evaluated, experimental use permits or similar must be obtained before investigations begin. Proprietary protection for evaluation of product formats still under development should follow industry specifications.

## Conclusion

Arboviruses transmitted by *Aedes* mosquitoes represent major international public-health concerns that will surely require a range of integrated interventions to be effectively controlled. As the scope of arboviruses continues to grow, development and evaluation of alternative vector-control products and strategies are critical to pursue. Following endorsement by global, national, and local authorities, effective strategies will have to be locally adapted to take into account the biology of the vector and virus transmission intensity, as well as human and financial resources. This review focuses on alternative strategies mainly for control of *A*. *aegypti* and *A*. *albopictus* because these two species are arguably the primary arbovirus vectors in the world. Alternative strategies will provide additional options for arbovirus control and potentially add value to existing strategies; however, until operational effectiveness and frameworks for use are in hand, further optimization of current strategies is warranted, to include innovative delivery methods of existing products (e.g., targeted indoor residual spraying [111]).

**Top five papers**

- **Context:** Moyes, Catherine L., et al. Contemporary status of insecticide resistance in the major *Aedes* vectors of arboviruses infecting humans. *PLoS Negl Trop Dis* ***1***11.7 (2017): e0005625. [96]
- **Novel larvicides and/or autodissemination:** Devine GJ, Perea EZ, Killeen GF, Stancil JD, Clark SJ, Morrison AC. Using adult mosquitoes to transfer insecticides to *Aedes aegypti* larval habitats. *Proceedings of the National Academy of Sciences*. (2009);106(28):11530–4. [17]
- **Spatial repellents:** Norris, Edmund J., and Joel R. Coats. Current and Future Repellent Technologies: The Potential of Spatial Repellents and Their Place in Mosquito-Borne Disease Control. *Int*. *J*. *Environ*. *Res*. *Public Health* (2017): 14(2), 124; 10.3390/ijerph14020124. [23]
- **Traps:** Barrera R, Acevedo V, Felix GE, Hemme RR, Vazquez J, Munoz JL, Amador M. Impact of autocidal gravid ovitraps on chikungunya virus incidence in *Aedes aegypti* (Diptera: Culicidae) in areas with and without traps. *Journal of medical entomology*. (2016);54(2):387–95. [112]
- **ATSBs:** Fiorenzano J. M., Philip G. Koehler P. G., Xue R-D. Attractive Toxic Sugar Bait (ATSB) For Control of Mosquitoes and Its Impact on Non-Target Organisms: A Review. *Int J Environ Res Public Health*. (2017); 14(4): 398. [113]
- **ITMs:** Banks, S. D., Murray, N., Wilder-Smith, A. and Logan, J. G. Insecticide-treated clothes for the control of vector-borne diseases: a review on effectiveness and safety. *Med Vet Entomol*. (2014): 28: 14–25. doi:10.1111/mve.12068. [55]
- **SIT/ RIDL:** Alphey L., McKemey A., Nimmo D., Oviedo M.N., Lacroix R., Matzen K., Beech C. Genetic control of *Aedes* mosquitoes. *Pathog Glob Health*. (2013); 107(4): 170–179. [114]
- ***Wolbachia*:** Iturbe‐Ormaetxe, I., Walker, T., and O'Neill, S. L. *Wolbachia* and the biological control of mosquito‐borne disease. *EMBO reports*. (2011); 12(6), 508–518. [79]
- **Gene drives:** Hammond A, Galizi R, Kyrou K, Simoni A, Siniscalchi C, Katsanos D, et al. A CRISPR-Cas9 gene drive system targeting female reproduction in the malaria mosquito vector *Anopheles gambiae*. *Nature biotechnology* (2016): 34(1): 78–83. [87]

**Key learning points**

- Arboviruses such as dengue, Zika, and chikungunya are reemerging worldwide with increasing prevalence and/or severity.
- Decades of efforts have failed to consistently control *Aedes* spp. mosquito populations and/or to curtail the cycle of arbovirus epidemics. Reasons are complex and include inadequate program implementation; lack of human, financial, and infrastructural capacity; IR; and inability to scale up existing interventions.
- Several alternative *Aedes* spp. arbovirus vector-control strategies are in the development pipeline and under evaluation, including biological and chemical approaches, but lack evidence with regards to when and where such strategies and/or products could offer greatest public health value and contribute to managing IR.
- Alternative strategies will need to be introduced using an approach of integrated vector management to include optimization of current methods, such as innovative delivery of existing products.
- An alternative vector-control strategy may be more useful via its role in integrated resistance management, even if it is of a similar efficacy to existing interventions.
