# Correction: Alternative strategies for mosquito-borne arbovirus control

**DOI:** 10.1371/journal.pntd.0007275

**Published:** 2019-03-26

**Authors:** Nicole L. Achee, John P. Grieco, Hassan Vatandoost, Gonçalo Seixas, Joao Pinto, Lee Ching-NG, Ademir J. Martins, Waraporn Juntarajumnong, Vincent Corbel, Clement Gouagna, Jean-Philippe David, James G. Logan, James Orsborne, Eric Marois, Gregor J. Devine, John Vontas

Figs 2 and 3 are incorrect. The image for Fig 2 is missing and has been incorrectly replaced with Fig 3. In addition, Fig 3 is in black and white and it should be in color. The authors have provided corrected versions of the figures and captions here.

**Fig 2:**
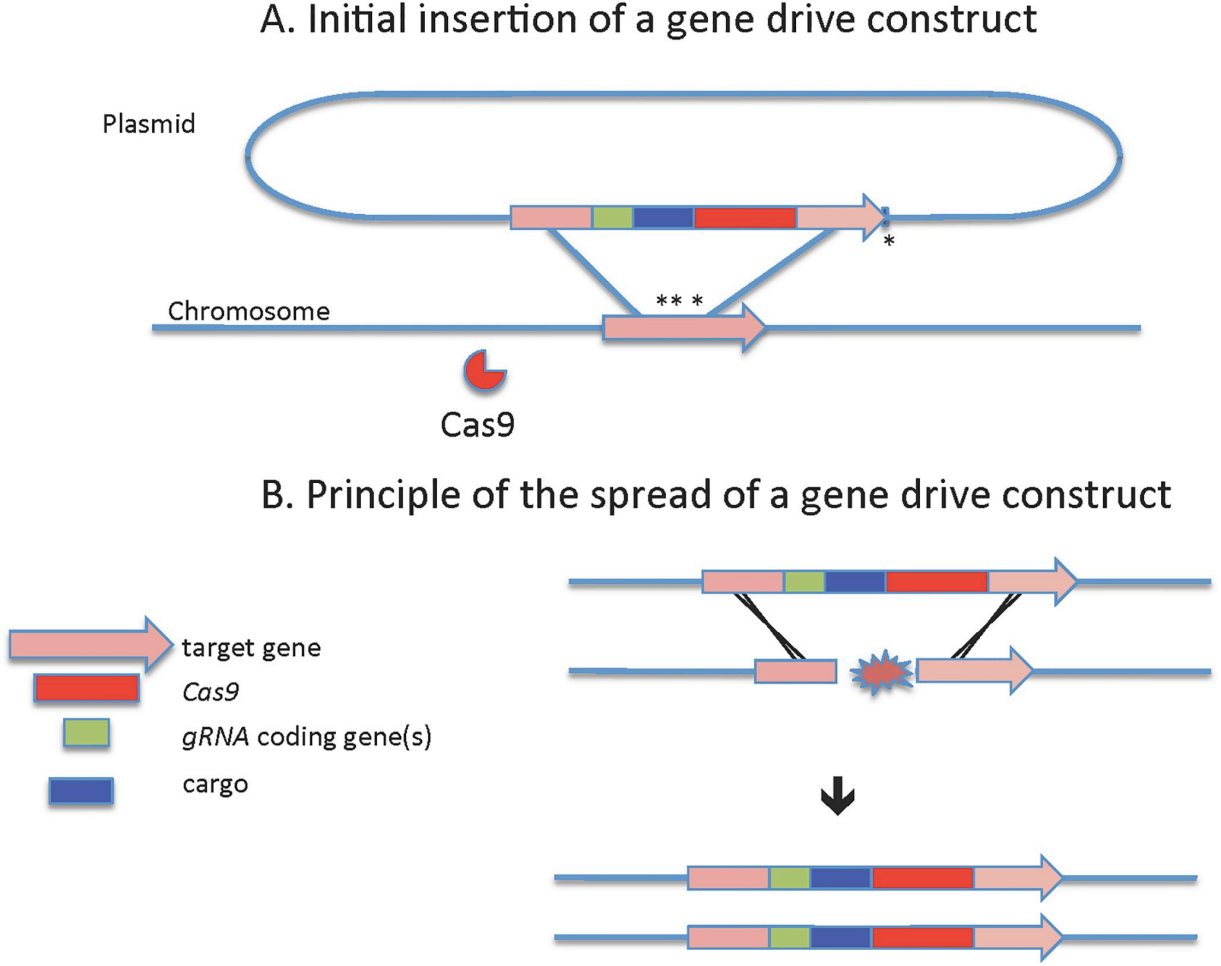
Principle of a gene drive. (A) Initial integration of a gene drive construct into the mosquito genome: Cas9 and the gRNAs encoded in the transgenic construct prepared as a plasmid can serve as molecular scissors mediating their own integration into the genomic target site they cut. Asterisks represent the cut sites determined by the gRNAs (three gRNAs in this example). Homologous recombination-mediated knock-in of the transgenic cassette occurs thanks to the target site flanking sequences cloned into the plasmid. (B) Spread of the gene drive in a mosquito population: mating between transgenic and nontransgenic mosquitoes places the transgenic construct in the presence of wild-type chromosomes that get cut by Cas9 at the target site determined by the gRNA(s). This break is repaired most frequently by homologous recombination with the intact chromosome, effectively copying the trans-gene into the broken wild-type chromosome and converting a heterozygous into homozygous cell. Cas9, CRISPR associated protein 9; gRNA, guide RNA.

**Fig 3:**
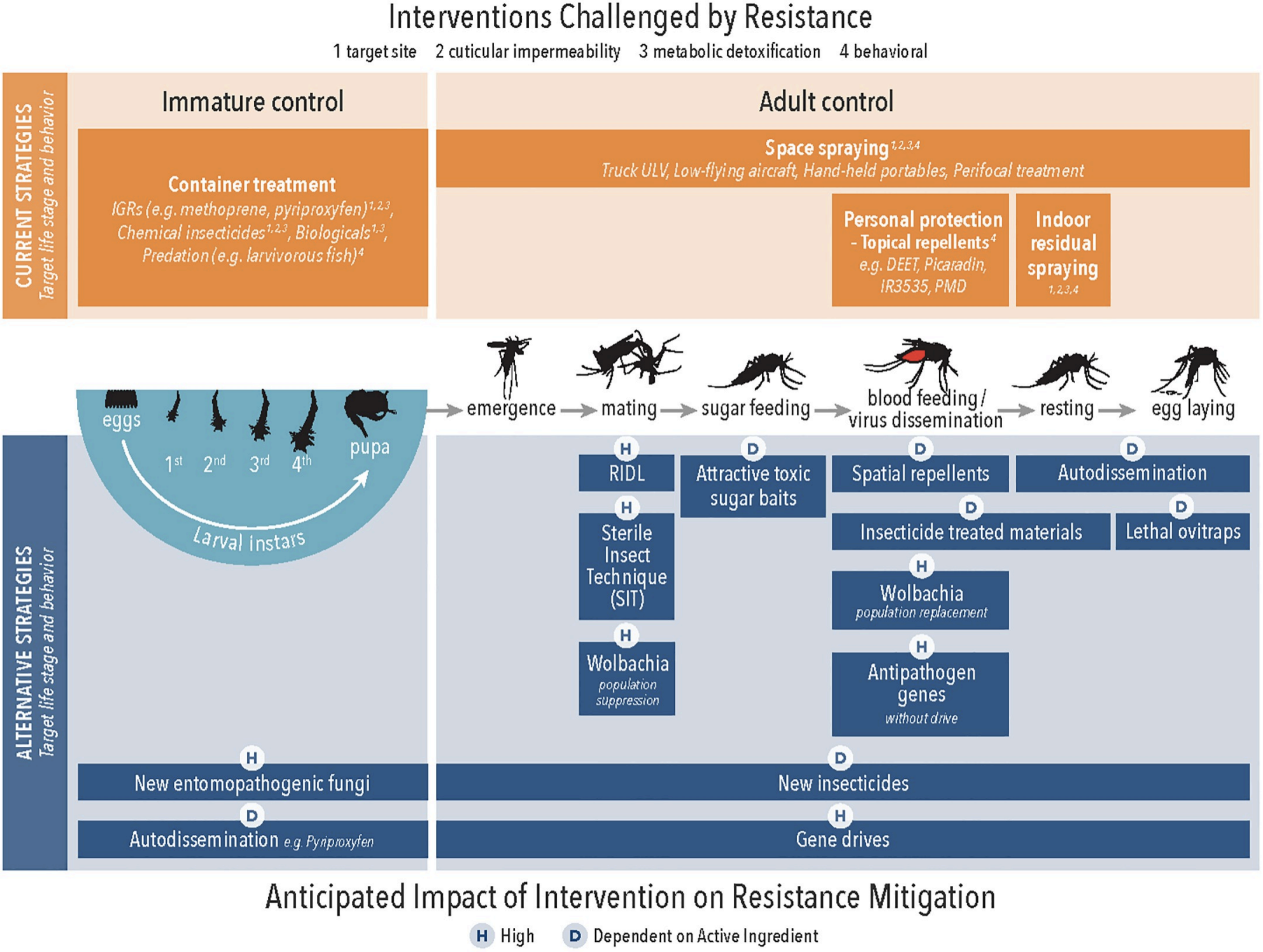
Current and alternative arbovirus control methods in the context of the targeted life stage of implementation and anticipated impact on IRM. IGR, insect growth regulator; IRM, insecticide resistance management; RIDL, release of insects with dominant lethality; ULV, Ultra-low volume spraying].
